# Supplementary material for: Learning curve and surgical outcome of robotic assisted colorectal surgery with ERAS program
Source: Sci Rep. 2022 Nov 29;12:20566. doi: 10.1038/s41598-022-24665-w (PMC9709162; doi:10.1038/s41598-022-24665-w)
Supplement: Supplementary file 2 — Supplementary Information 2. [file 41598_2022_24665_MOESM2_ESM.zip › RAL Raw data-Ñ[▒K/068/HS_ñw╝╨░O▒Kñσ.pdf]

正式摘要(電子簽章：000000213950995)

W55 / W55 ☐英文標題(注意:無英文姓名-請補病人基本檔!) ☐【讀取】憑證

臺中榮民總醫院

出院病歷摘要

轉入號： 轉出號：

[redacted]

(8)流水編號	
(9)入院日期	110/03/30 (2021) 直腸外科 W55 - 242
(10)轉科日期	
(11)出院日期	110/04/07 (2021) 住院天數計 8 日

診斷

- 入院 :1. rectum ca s/p CCRT
- 出院 :1. Rectum CA, 5cm FAV, cT3N1bM0, Stage IIIA
  CCRT(54Gy/30frs, 5-FU+LV 2020/12/14, 12/21, 1/4, 1/18, 1/25, 2/9),
  with anal skin erosion s/p EGF 2021/2/1
  s/p FOLFOX 2/23 3/9
  s/p robotic LAR TME on 2021/3/31
  2. BPH s/p green light laser on 2021/2/5
  3. Cystic lesion of lung, RLL, r/o lung cancer

主訴

- For scheduled robotic LAR.

病史

- The 65 year old male had history of
  1. RECTAL CA, 5CM FAV, c T3 N0 M0, Stage IIA
  under CCRT (54Gy/30frs, 5-FU+LV 12/14, 12/21, 1/4, 1/18, 1/25, 2/9) since 2020/12/14-2/3
  with anal skin erosion s/p EGF 2/1
  2. BPH WITH BOO s/p Green Light，Laser phote-vaporization of theprostate Transurethral biops on 2021/02/05
  3. Cystic lesion of lung, RLL, r/o lung cancer
  There was chronic anal pain for long time and aggravated pain with mudy stool passage in recent one week, then he came to our ER for help on 3/23. According to coloscopy on 3/23 showed that up to 10 cm FAV, the colon tumor gets smaller and anus ulcer. Dr. Lin arranged restaging by MRI, whiched showed rectal cancer under CCRT, stable as compared with previous MRI 2021/1/14. Robot Assisted Low Anterior Resection was arranged on 3/31. Under the impression of rectal cancer, he was admitted for surgical intervention and further treatment.

體檢發現

- CEA: 2.8(2020/12)--2.8(2021/1)
  2020/11 CT: no mets.
  2020/12 PET: uptake at the rectum (SUVmax:9.4/1h)
  2021/1 MRI: rectum ca. Lung CT: RLL mass.
  - General appearance :
    - easy looking , conscious clear , without cardiopulmonary distress
    - Vital signs自動帶入date 體溫(BT) 脈搏(P) 呼吸(R) 血壓(BP) 身高(BH) 體重(BW) BMI
      20210330 36.9C 96/min 18/min 132/87mmHg 168.5cm 78.6Kg 27.7
    - Vital signs: B.P. 132/87 mmHg , P.R. 96 /min , R.R. 18 /min , B.T. 36.9 ℃
  - HEENT :
    - Eyes: no anemic conjunctiva, no icteric sclera, normal EOM
    - ENT: no otorrhea, no rhinorrhea, no erythematous mucosa, no injected tonsil, no deformity
  - Neck: supple, freely movable, trachea midline,no deformity, no engorged jugular vein , no palpable thyroid or lymph node enlargement
  - Chest:
    - Inspection: Grossly normal, symmetric and fully expansion, no accessory muscle used, no operation scar, no spider angiomata, no gynecomastia
    - Palpation: no palpable mass or nodule, no subcutaneous emphysema, no tenderness
    - Ausculation: clear breathing sound
  - Heart:
    - Palpation: no thrill, no heave
    - Percussion: normal heart size
    - Ausculation: regular heart beat, normal S1, S2, no gallop, no murmur
  - Extremities: No deformity, full ranged of motion without limitation, no clubbing finger or nail cyanosis, no pitting edema, no ulcer or pigmentation, no obvious skin lesion, no varicose vein, no bone, joint or muscle tenderness

CRS CONDITION

- Karnofsky performance status: (100) Normal, no complaints, no evidence of disease
- PE of Abdomen:

- CEA: 2.8 ng/ml
- Imaging Studies:
- Colonoscope report: up to 10 cm FAV, the colon tumor gets smaller and anus ulcer
- Figures:
  1. colon & rectum

手術日期及方法

- 手術日期及方法20210331 Robot Assisted Low Anterior Resection

住院治療經過

- The patient was admitted for robotic-assisted LAR after MRI restaging of his rectal cancer on 2021/3/21. During hospitalization, pre-operative survey showed acceptable conditions. He underwent surgery on 2021/3/31. After surgery, he went back to ward for post-operative care. He started perioperative coaching training after surgery. He became feverish on 4/1 without any discomforts. His fever subsided in the next day. Under relatively stable condition, he was therefore dischargeon 4/7 with OPD follow-up.

合併症

一般檢查

- 累積報告 :

DATE	TIME	ALB	TP	BIL.T	BIL.D	ALKP	AST	ALT	LDH	CHOL	TG	HDL-C	LDL	UA	NH3	AMY
1100319	2140			0.68				26								
1100319	2141													4.7		
1100322	0901									160		40				
1100330	1728	3.8	5.9	0.43	0.11		27	36	200							
1100331	1546	3.6		0.73			25	29								

DATE	TIME	WBC	RBC	HGB	HCT	MCV	PLT	BLST	NEUT	BAND	SEG	LYM	MON	EOS	BAS
1100319	2139	7640		12.3			251				75.5	10.6	8.2	5.0	0.7
1100322	0901	6110	3.80	12.5	37.0	97.4	243		68.8			18.5	8.2	3.8	0.7
1100331	1547	10080		12.4	35.2		217		93.2			4.2	2.4	0.1	0.1
1100406	0944						261		78.9			6.6	6.9	7.3	0.3
1100406	0946	5940	3.49	11.5	34.4	98.6									

DATE	TIME	NA	K	CL	CA	BUN	CREAT	Mg	P	CRP	ESR	PCT	LIPASE	LACTATE
1100319	2140	137	3.7		8.6	12	0.82			0.084				
1100330	1728	140	4.1	106	8.7	15	0.89							
1100331	1546	138	3.8		8.6	12	0.76							
1100406	0844													6.6
1100406	0913	139				8	0.82							

DATE	TIME	GLUCOSE	PAB	TRF	ZN	CU	KETONE	CK	CKMB	PT-P	PT-C	APTT-P	APTT-C	TRO-T
1100319	2140									10.2	10.6	25.3		
1100330	1729	89												

DATE	TIME	PH	BEB	SCVO2	PO2	HCO3	HGB	PCO2	TCO2
1100319	2139	7.413	1.2	89.3	56.1	26.0		41.6	27.2

特殊檢查

病理報告

- pending.

其它

出院時情況

- 3.改門診治療

出院指示

藥名	劑量	劑量單位	用法	頻次	數量	數量單位	現況	天數	單位
25mg Utapine tab	0.5	TAB	PO	BID	7	TAB	TH	7	天
(□)Takepron OD tab 30mg	1	TAB	PO	QDAC	7	TAB	TH	7	天
Mag. Oxide tab	2	TAB	PO	TID	42	TAB	TH	7	天
Ultracet tab	1	TAB	PO	Q6HPRN	12	TAB	TH	3	天

出院計劃

- 門診□期
  - date on 2021/04/13

主治醫師 2222H 林俊余 / 住院醫師 9958C 林強生

正式摘要(電子簽章：000000213950995)

W55 / W55

doctorRank:B
